# Basophil activation test and lymphocyte transformation test in cefuroxime-induced anaphylactic reactions

**DOI:** 10.3389/falgy.2025.1532775

**Published:** 2025-03-27

**Authors:** Andreas Glässner, Diana Dubrall, Gerda Wurpts, Philipp Deck, Günther Weindl, Caspar A. Heubach, Amir S. Yazdi, Bernhardt Sachs

**Affiliations:** ^1^ Research Division, Federal Institute for Drugs and Medical Devices (BfArM), Bonn, Germany; ^2^ Institute for Medical Biometry, Informatics and Epidemiology, University Hospital of Bonn, Bonn, Germany; ^3^ Department of Dermatology and Allergology, RWTH University Hospital Aachen, Aachen, Germany; ^4^ Aachen Comprehensive Allergy Center (ACAC), University Hospital Aachen, Aachen, Germany; ^5^ Pharmacology and Toxicology, Pharmaceutical Institute, University of Bonn, Bonn, Germany

**Keywords:** cefuroxime, anaphylactic reaction, lymphocyte transformation test, basophil activation test, spontaneous reports

## Abstract

**Introduction:**

Cefuroxime allergy may present as a delayed-type reaction or as an immunoglobulin (Ig)E-mediated immediate-type anaphylactic reaction. The basophil activation test (BAT) is a diagnostic tool for cefuroxime-induced immediate-type reactions, whereas the lymphocyte transformation test (LTT) is typically applied in delayed-type drug allergy. This study aimed to compare the results of the BAT and LTT in 15 patients with cefuroxime-induced anaphylactic reactions considered as confirmed. The pharmacoepidemiological part aimed to analyze spontaneous reports of cefuroxime-associated anaphylactic reactions in the European adverse drug reaction database (EudraVigilance).

**Methods:**

In EudraVigilance, 668 reports of cefuroxime-associated anaphylactic reactions for the European Economic Area (EEA) between 2010 and 2023 were analyzed, with 182 (27.2%) of these reports originating from Germany. The BAT and the LTT were performed according to standard protocols. Except for one patient, all BAT were performed prior to the skin tests, whereas all LTT were performed thereafter.

**Results:**

Almost all reports were classified as serious (EEA, 99.3%; Germany, 98.9%). In 60.8% (EEA) and 66.9% (Germany) of reports with respective information, the reaction occurred after intravenous administration. BAT was performed in 12 of 15 patients (3/12 positive; sensitivity 25%), while LTT was performed in all 15 patients (7/15 positive; sensitivity 46.7%).

**Conclusions:**

Our analysis highlights the importance of cefuroxime-associated anaphylactic reactions, as almost all of the spontaneous reports were classified as serious. Neither a negative BAT nor LTT can rule out a sensitization in cefuroxime-induced anaphylactic reactions.

## 1 Introduction

Cefuroxime is one of the most commonly used cephalosporins in clinical practice. It is available for intravenous and oral use (prodrug cefuroxime axetil) (1).

Cefuroxime allergy may present either as a delayed-type reaction like maculopapular exanthem or as an immunoglobulin (Ig)E-mediated immediate-type anaphylactic reaction with severities ranging from grade I (e.g., urticaria) to grade IV (e.g., potentially fatal cardiovascular arrest) (2).

The diagnosis of cefuroxime allergy is based on (i) the medical history, (ii) the clinical phenotype of the reaction, (iii) *in vitro* tests including the basophil activation test (BAT), (iv) *in vivo* skin tests (prick and intracutaneous), and (v) provocation tests if necessary and appropriate (3). No commercial kit is available for the *in vitro* detection of cefuroxime-specific IgE in Germany. Therefore, the BAT is frequently used in cefuroxime-induced immediate-type reactions. In accordance with respective guidelines (1, 3), in patients with anaphylactic reactions ≥grade II, the BAT—as an *in vitro* test—is performed prior to the *in vivo* tests for safety reasons.

The BAT is based on allergen-specific activation of basophil granulocytes (4). Hence, its application is limited to immediate-type reactions that are mediated by the adaptive immune system (i.e., IgE) or by other mechanisms not involving the specific immune system (e.g., direct degranulation of basophils) (5). For beta-lactam antibiotics, a sensitivity of 30%–50% is reported, with corresponding specificities ranging from 80% to 100% (5). To ensure optimal sensitivity, it should be conducted within 1 year after the assumed allergic reaction (6).

The lymphocyte transformation test (LTT) is typically applied in delayed-type drug allergy where the distal effector phase is mediated by T cells. For delayed-type reactions to beta-lactams, a sensitivity and specificity of 49.1% and 94.6% were reported, respectively (7). The LTT is also helpful in IgE-mediated allergic reactions (8), albeit with a lesser sensitivity (9, 10). However, one study reported an even better sensitivity (11). The explanation given is that the LTT detects drug-specific memory T cells, which are a common starting point in the initial sensitization irrespective of its distal mediation (IgE on mast cells/basophils or T effector cells) (12).

The LTT should be performed optimally after the acute phase and within 1 year after the reaction. Due to its complexity, it is currently not part of the routine diagnostic (8, 10, 11). The read-out parameter of the classical LTT is proliferation, based on the determination of radioactive 3H-thymidine incorporated into proliferating lymphocytes. In recent years, the measurement of cytokines by enzyme-linked immunosorbent assay (ELISA) or ELISPOT has been used increasingly as a read-out parameter (13). Since cytokine detection appears to be superior in terms of sensitivity (10), it was also used in this analysis.

The objective of the present analysis was to compare the results of the BAT and LTT among patients with cefuroxime-induced anaphylactic reactions. Notably, we selected patients with a stringent medical history of a cefuroxime-induced anaphylactic reaction (causal association probably or certain according to the WHO classification 14). In this patient group, we considered the cefuroxime allergy as confirmed. Hence, it should ideally be detectable in the BAT and LTT.

We combined this experimental approach with a pharmacoepidemiological analysis of the spontaneous reports of cefuroxime-associated anaphylactic reactions in Germany and the European Economic Area (EEA). This approach aimed to highlight the importance of cefuroxime anaphylactic reactions in clinical practice by evaluating the number of reports over time and their seriousness. In addition, in the pharmacological part, the type of anaphylactic symptoms that have occurred could be characterized more deeply to gain further information on the severity of the reactions.

## 2 Materials and methods

### 2.1 Pharmacoepidemiological part

#### 2.1.1 Spontaneous reports, EudraVigilance, and prescription data

Adverse drug reactions (ADRs) can be reported spontaneously by healthcare professionals or non-healthcare professionals in everyday practice (spontaneous reports). A more detailed description can be found elsewhere (16).

EudraVigilance is the ADR database of the European Medicines Agency and includes all spontaneously reported ADRs from the member states of the EEA (17). In EudraVigilance, the coding of ADRs and drugs is based on MedDRA terminology (18) and the EudraVigilance medicinal product dictionary (19), respectively.

We extracted all spontaneous reports from the EEA received between 2010 and 2023, in which cefuroxime was reported as suspected/interacting (*n* = 5,648). Subsequently, reports describing anaphylactic reactions were identified by applying the standardized MedDRA query “anaphylactic reaction (narrow)” (*n* = 668, 11.8%) (18). Overall, 182 (27.2%) of the reports were from Germany.

Cefuroxime prescription data were queried via the public dashboard of PharMAAnalyst (20). The number of drug prescriptions represents the total number of *outpatient* prescriptions for patients with statutory health insurance [almost 90% of the German population (21)] dispensed in German pharmacies between 2012 and 2022. *Inpatient* prescriptions are not covered, and respective data are not available.

The annual number of spontaneous reports of cefuroxime-associated anaphylactic reactions was divided by the annual number of cefuroxime prescriptions to calculate so-called *reporting rates* per year. These are presented as the number of reports of cefuroxime-associated anaphylactic reactions per 1 million prescriptions.

##### 2.1.1.1 Performed analyses

The 668 and 182 reports of cefuroxime-associated anaphylactic reactions from the EEA and Germany, respectively, were descriptively analyzed with regard to the reported indications for cefuroxime therapy, severity criteria, and symptoms on the level of the system organ classes (22). In the latter, we focused on the number of reports for the system organ classes that are used for the classification of the severity of anaphylactic reactions according to Ring and Messmer (23). More than one of these system organ classes can be reported per ADR report.

Descriptive statistical analyses were performed for the pharmacoepidemiological and experimental analyses.

### 2.2 Experimental part

#### 2.2.1 Patients

In this study, we included 15 patients with cefuroxime-induced anaphylactic reactions ranging from grade I to IV who were recruited from the Department of Dermatology and Allergology of the RWTH Aachen University Hospital. The results of three patients have already been reported elsewhere in a different context (9). The allergological work-up was performed according to respective guidelines (1, 3). Notably, as described above, for safety reasons a BAT is a standard performed prior to skin testing (in particular in patients with higher-grade anaphylactic reactions). If the BAT is positive, no skin tests will be performed in patients with cefuroxime-induced anaphylactic reactions.

In keeping with the data protection rules, we could not examine whether these 15 patients were among those 182 patients from Germany for which an ADR report was filed.

#### 2.2.2 BAT and LTT

The LTT was performed as described previously (24, 25). In brief, PBMC were isolated from the donor blood samples by density gradient centrifugation and resuspended in RPMI 1640 medium supplemented with 5% autologous plasma, MEM non-essential amino acid solution (100×, Thermo Fisher Scientific), and 1 mM sodium pyruvate (Thermo Fisher Scientific). A total of 5 × 10^5^ cells in a final volume of 200 µl were seeded in 96-well round bottom plates and incubated with either cefuroxime at two concentrations (50 and 200 µg/ml) (8), tetanus toxoid (0.1 µg/ml, positive control), or RPMI medium as an unstimulated reference. For each condition, a total of six replicates were prepared and incubated for 6 days. Subsequently, the supernatant for an individual condition was pooled and stored at −80°C prior to ELISA measurements. IFN-y and IL-5 secretion was determined by ELISA as read-out (Biolegend, USA) (25). A stimulation index (SI) (IFN-y/IL-5 secretion stimulated/unstimulated cultures) >3 was considered as positive.

The BAT was performed according to the manufacturer's recommendations for the Flow Cast including two different positive controls (anti-FceRI mAb and fMLP). As outlined in the Flow Cast product information, the sample was considered evaluable if one of those two controls induced activation of >10% of basophils (26). For cefuroxime, the BAT was considered positive, if more than 5% of the CCR3-positive basophiles expressed the activation marker CD63 on the surface. Among the 11 patients tested in the BAT, there was no non-responder (<10% basophils following stimulation with the aforementioned positive controls; 26). All BAT were performed prior to the skin tests except for one patient (no. 10). All LTT were performed after the skin tests except for Patient 8. Patient 10 had been tested negative for cefuroxime in the skin test previously and had been exposed to cefuroxime without a reaction. The patient later developed an anaphylactic reaction to cefuroxime and was then skin tested positive for cefuroxime. Thereafter, the BAT was performed as an additional diagnostic measure.

The LTT was performed within 1 year after the allergic reaction in 10/15 patients, and after 12, 13, 20, 25, and 53 months in 5/15 patients, respectively (mean, 11.8 months; SD, 13 months). The BAT was conducted within one year in all patients. The earliest time of conductance of the LTT and BAT was 0.5 months after the reaction in Patient 8 (see Table 1).

**Table 1:** Patient characteristics and results of the skin tests, BAT, and LTT.

Number of patients	Age	Sex	Grade of anaphylactic reaction^b^	Intracutaneous test	Prick test	BAT	Total IgE	LTT-ELISA (IFN-γ/IL-5) [SI]	Time to testing (LTT)	Time to testing (BAT)	Time to testing (skin test)
1^a^	52	F	Grade I	Positive	Negative	Negative	97.5 kU/L	Negative	5 months	0.5 month	1 month
2	56	F	Grade III	n.d.	Positive	Negative	54.8 kU/L	Positive (IFN-γ: 7.5)	2 months	1 month	2 months
3	56	F	Grade III	n.d.	Positive	Negative	54.8 kU/L	Positive (IFN-γ: 3.3)	4 months	3 months	4 months
4	48	F	Grade IV	Positive	Unclear	Negative	274 kU/L	Positive (IFN-γ: 6.7)	25 months	10 months	11 months
5	38	F	Grade II	n.d.	n.d.	Positive (22% CD63+)	17.0 kU/L	Positive (IFN-γ: 32.5)	9 months	8 months	n/a
6	64	M	Grade III	n.d.	n.d.	Positive (48% CD63+)	n.d.	Negative	7 months	2 months	n/a
7	78	F	Grade III	n.d.	Positive	Negative	53.2 kU/L	Negative	4 months	2 months	2 months
8	46	F	Grade III	n.d.	Positive	Negative	n.d.	Negative	0.5 month	0.5 month	4 months
9	58	F	Grade III	Positive	n.d.	Negative	63.2 kU/L	Positive (IFN-γ: 35.7)	20 month	3 months	10 months
10^c^	63	F	Grade II–III	n.d.	positive	Negative	15.4 kU/L	Negative	13 months	10 months	4 months
11	58	F	Grade III	n.d.	Positive	n.d.	62.0 kU/L	Negative	8 months	n.d.	4 months
12	61	F	Grade I	n.d.	Positive	n.d.	n.d.	Positive (IFN-γ: 3.5)	6 months	n.d.	4 months
13	57	M	Grade I	n.d.	Positive	n.d.	n.d.	Negative	53 months	n.d.	51 months
14	62	F	Grade III–IV	Negative	Negative	Positive (46% CD63+)	91.2 kU/L	Positive (IFN-γ: 3.9)	12 months	1 month	2 months
15	38	M	Grade II	Negative	Positive	Negative	145 kU/L	Negative	9 months	3 months	5 months

BAT, basophile activation test; LTT, lymphocyte transformation test; n.d., not determined; n.a., not applicable.

^a^ For Patient 1, an LTT with radioactive read-out (SI: 3.2) was performed by an external laboratory.

^b^ According to Ring and Messmer (22).

^c^ Skin test performed 4 days prior to BAT.

PBMC from a control person with no known sensitization to cefuroxime was included in each LTT experiment. Since the BAT was performed in the routine diagnostic, no results from control persons in the same experiment were available.

### 2.3 Ethics statement

The experimental part of the study was approved by the ethics committee of the RWTH Aachen University Hospital (study number EK 309/19) and the North Rhine medical council (study number: 2020098). All donors signed an appropriate informed consent form. The pharmacoepidemiological part of the study was approved by the ethics committee of the University Hospital Bonn (study number 458/20).

## 3 Results

### 3.1 Pharmacoepidemiological part

**Figure 1:**
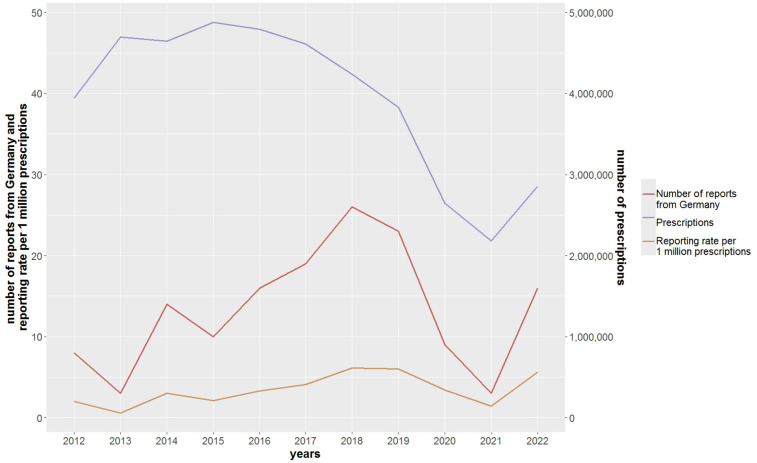
Number of spontaneous reports of cefuroxime-associated anaphylactic reactions and their reporting rates and the number of cefuroxime outpatient prescriptions in Germany.

An increase in the number of spontaneous reports of cefuroxime-associated anaphylactic reactions was observed for Germany (Figure 1) and the EEA, especially between 2016 and 2019. In more than half of the reports with respective information (EEA, 60.8%; Germany, 66.9%), the anaphylactic reaction occurred after intravenous administration and in 34.8% (EEA) and 31.8% (Germany) after oral administration (see Table 2). While the number of outpatient prescriptions of cefuroxime in Germany decreased substantially, the reporting rates (number of spontaneous reports/number of outpatient prescriptions) increased slightly (except for 2021).

**Table 2:** Descriptive analyses of indications, seriousness criteria, and system organ classes reported in spontaneous reports of cefuroxime-associated anaphylactic reactions originating from the EEA and Germany.

	Reports of cefuroxime-associated anaphylactic reactions from the EEA (*n* = 668)	Reports of cefuroxime-associated anaphylactic reactions from Germany (*n* = 182)
Number of reports with more than one drug reported as		
Suspected/interacting	19.9% (*n* = 133)	12.6% (*n* = 23)
Suspected/interacting and concomitant	47.8% (*n* = 319)	45.6% (*n* = 83)
Three most frequently reported indications on HLGT level of MedDRA terminology^a^		
Information reported	71.7% (*n* = 479)	71.4% (*n* = 130)
Therapeutic procedures and supportive care (prophylaxis)		
Infections—pathogen unspecified	42.0% (201/479)	51.5% (67/130)
Bacterial infectious disorders	33.8% (162/479)	26.9% (35/130)
	4.6% (22/479)	3.1% (4/130)
The most frequently reported routes of administration		
Number of cefuroxime applications	670^b^	183^b^
Number of applications with information	79.3% (*n* = 531)	82.5% (*n* = 151)
Intravenous	60.8% (323/531)	66.9% (101/151)
Oral	34.8% (185/531)	31.8% (48/151)
Others	4.3% (23/531)	1.3% (2/151)
Classification of seriousness^c^		
Serious	99.3% (*n* = 663)	98.9% (*n* = 180)
Death	5.5% (*n* = 37)	8.2% (*n* = 15)
Life-threatening	55.4% (*n* = 370)	56.6% (*n* = 103)
Hospitalization/prolongation thereof	43.9% (*n* = 293)	48.4% (*n* = 88)
Number of system organ classes^d^ reported considered by Ring and Messmer^e^		
Number of reports related to the four system		
Organ classes	40.4% (*n* = 270)	53.8% (*n* = 98)
One organ system	44.1% (119/270)	41.8% (41/98)
Two organ systems	40.7% (110/270)	46.9% (46/98)
Three organ systems	12.2% (33/270)	7.1% (7/98)
Four organ systems	3.0% (8/270)	4.1% (4/98)
Number of reports with regard to the system organ classes^d^ which form the basis for the classification by Ring and Messmer^e^		
Skin and subcutaneous disorders	55.9% (151/270)	48.0% (47/98)
Gastrointestinal disorders	28.9%% (78/270)	24.5% (24/98)
Respiratory disorders	53.0% (143/270)	52.0% (51/98)
Cardiovascular disorders	36.3% (98/270)	49.0% (48/98)
Number of reports with combinations of at least two of the system organ classes^d^ considered by Ring and Messmer^e^		
Skin and subcutaneous and gastrointestinal disorders	18.5% (50/270)	13.3% (13/98)
Skin and subcutaneous and respiratory disorders	27.0% (73/270)	20.4% (20/98)
Skin and subcutaneous and cardiac disorders	13.7% (37/270)	17.3% (17/98)
Gastrointestinal and respiratory disorders	13.3% (36/270)	9.2% (9/98)
Gastrointestinal and cardiac disorders	5.9% (16/270)	7.1% (7/98)
Respiratory and cardiac disorders	16.7% (45/270)	25.5% (25/98)
Number of reports with combinations of at least three of the system organ classes^d^ considered by Ring and Messmer^e^		
Skin and subcutaneous and gastrointestinal and respiratory disorders	9.6% (26/270)	6.1% (6/98)
Skin and subcutaneous and gastrointestinal and cardiac disorders	3.3% (9/270)	4.1% (4/98)
Skin and subcutaneous and respiratory and cardiac disorders	7.0% (19/270)	8.2% (8/98)
Gastrointestinal and respiratory and cardiac disorders	4.1% (22/270)	5.1% (5/98)

^a^ MedDRA terminology consists of five different hierarchical levels (18).

^b^ Two patients in the EU reports and one patient in the reports from Germany received cefuroxime orally and intravenously resulting in 670 and 183 applications, respectively.

^c^ The classification of seriousness follows the legal definition described in (15).

^d^ More than one ADR of the respective system organ class can be reported per ADR report. Furthermore, more than one system organ class can be reported per ADR report. In some ADR reports, only the diagnosis of anaphylactic reaction or anaphylactic shock may be reported and these reports cannot be assigned to any of the four system organ classes.

^e^ According to Ring and Messmer (23).

Almost all of these spontaneous reports were classified as serious (EEA, 99.3%; Germany, 98.8%), and slightly more than half of them were designated as life-threatening (EEA, 55.4%; Germany, 56.6%). Fatal outcome was mentioned in 5.5% (EEA) and 8.2% (Germany), respectively.

In those reports with respective information (EEA, 71.7%; Germany, 71.4%), the most frequently reported indication was antibiotic prophylaxis (EEA, 42.0%; Germany, 51.5%).

Moreover, 40.4% of the EEA and 53.8% of the reports from Germany reported symptoms of the system organ classes, which form the basis for the classification by Ring and Messmer (Table 2). Most of these reports included symptoms of one or two of these organ systems (EEA, 84.8%; Germany, 88.7%). Of these ADR reports, respiratory disorders were reported in 53.0% of the EEA and 52.0% of the reports from Germany. Cardiac disorders were mentioned in 36.3% (EEA) and 49.0% (Germany). The evaluation of the co-occurrence of several system organ classes is shown in Table 2.

### 3.2 Experimental part

Out of the 15 patients, 12 were female, and 3 were male. The mean age was 55.7. An LTT was performed in all patients, but a BAT was not performed in three patients.

The BAT was positive in 3/12 patients compared to 7/15 patients in the LTT (sensitivity BAT 25% and LTT 46.7%) (see Table 1). Four control persons with no reported clinical history (note: no additional skin tests were performed in control persons) of sensitization to cefuroxime gave a (false) positive result in the LTT (specificity 73.3%, PPV = 0.70, NPV = 0.60).

The LTT with IL-5 as read-out was negative in all 13 patients and controls. Notably, PBMC from all patients and controls showed positive IL-5 and IFN-y responses to the positive control tetanus toxoid, confirming the technical validity of both tests.

Skin tests (prick and/or intracutaneous) were performed in 13/15 patients and were positive (prick and/or intracutaneous) in 11/13 cases. In the two patients with negative skin tests, a cefuroxime-induced anaphylactic reaction was still considered confirmed due to the very short time of onset of the reaction following administration of cefuroxime (5 min and immediately, respectively).

## 4 Discussion

### 4.1 Pharmacoepidemiological part

The pharmacoepidemiological part of our study underlines the clinical relevance of cefuroxime-associated anaphylactic reactions since (i) almost all of the anaphylactic reactions were classified as serious and (ii) the number of reports increased in the past. Although anaphylactic reactions in general can take a serious course (3, 23), we consider the number of spontaneous reports classified as serious to be remarkably high (Germany, 98.9%; EEA, 99.3%). Accordingly, involvement of the respiratory tract and cardiovascular system was observed in about half of the reports from Germany with respective information. Differences in the reporting behaviors between several countries could be the reason for the difference in the proportion of patients with cardiovascular ADRs in the ADR reports from the EEA (36.3%) compared to Germany (49.0%).

The increase in the reporting rates of cefuroxime-associated anaphylactic reactions over time (2013–2019 and 2021–2022) may reflect an increased inpatient use, a higher awareness of ADR reporting in general, changes in reporting obligations (27), or other so far unknown factors (16). The lower number of reports in 2020 and 2021 may be related to a preferential reporting of COVID-19 vaccines instead of ADRs related to other drugs as observed in other studies (28). Our calculated *outpatient* reporting rate is subject to underreporting (29) concerning the numerator since not all anaphylactic reactions are reported, thereby underestimating the real incidence. On the other hand, the denominator does not include *inpatient* cefuroxime administrations [not (publicly) available], thereby overestimating the real incidence. The real incidences cannot be determined based on spontaneous reports (16, 29).

In line with the high proportion of intravenous administration, antibiotic prophylaxis, e.g., in the context of surgery, was the indication most frequently reported. However, cefuroxime-associated anaphylactic reactions might also be more often detected, and therefore reported, in the context of surgery than in other settings. Notably, in the spontaneous reports, cefuroxime was considered suspected or interacting and was the only drug reported as suspected/interacting in the majority of the reports. However, other drugs administered concomitantly may also have contributed to or caused the anaphylactic reaction. Since no individual assessment of the ADR reports, e.g., with regard to the causal relationship, has been performed, the number of spontaneous reports in which cefuroxime was considered the only causal drug (among other drugs given) cannot be determined.

### 4.2 Experimental part

The timing of the LTT and BAT could, to some extent, explain why the LTT performed better than the BAT (46.7% vs. 25% sensitivity, respectively). While the BAT was performed prior to the skin tests in 11/12 patients, the LTT was always conducted thereafter except for one patient. Therefore, an immunological booster effect may have occurred in these patients before the LTT except for Patient 8 who tested negative in the LTT. However, it does not explain why the BAT proved negative in 75% of the patients. The specificity and sensitivity of the BAT with cephalosporins or beta-lactam antibiotics in general vary over a broad range from a high specificity of 92.3% but a low overall sensitivity of 20.8% (30) to a sensitivity of around 60% (1). The sensitivity and specificity of BAT can vary depending on factors such as the specific drug, test protocol, and patient population. For cefuroxime specifically, more targeted research would be needed to determine its precise sensitivities and specificity values in BAT. None of the 12 patients tested in the BAT was a non-responder in accordance with the specifications in the product information of the manufacturer (see Section 2.2.2) (26). This absence of a non-responder in 12 patients is compatible with the information given in the product information of the Flow Cast. There it is stated that 6.1% (out of *n* = 98) were non-responders to anti-FceRI mAb and 4.9% (out of *n* = 61) to fMLP (26).

Notably, in Patient 8, BAT and LTT were performed 0.5 months after the reaction with negative results in both tests and 3.5 months prior to skin testing (positive). It may be discussed that the time to testing was too short for both tests in this particular patient. However, since the negative results accounted for both tests, the exclusion of this patient would not substantially favor one of the tests with regard to the sensitivity [BAT 27.3% (3 pos./11 pat. tested); LTT 50% (7 pos./14 pat. tested)].

The modest sensitivity of the LTT (46.7%) in these patients with immediate-type reactions complies with the respective findings in literature. As stated above, the LTT in principle may also be used for immediate-type reactions (8), albeit with a lower sensitivity (9, 10).

Interestingly, we did not observe any IL-5 secretion of the PBMC of the patients upon cefuroxime stimulation, although one could expect the secretion of this Th2 cytokine since all patients developed an immediate (Th2)-type reaction by the clinical phenotype. However, it has been described in the literature that the *in vivo* phenotype of the reaction does not need to correspond to the *in vitro* phenotype (31).

### 4.3 Implications for clinical practice

Physicians should be aware that cefuroxime, in addition to delayed-type reactions, also induces severe anaphylactic reactions, in particular following intravenous application. Hence, monitoring patients receiving cefuroxime intravenously is important to avoid severe courses of anaphylactic reactions by early interventions. In addition, taking a break between the administration of cefuroxime before surgery and the administration of other drugs (e.g., muscle-relaxing drugs) may facilitate the identification of the causative drug based on the timing of a potential reaction. Finally, neither a negative BAT nor LTT can rule out a sensitization in cefuroxime-induced anaphylactic reactions.

Our results may trigger further studies specifically designed to compare the performance of the LTT to the BAT in immediate-type reactions induced by other drugs. Likewise, the impact of skin testing with the suspected drug before the BAT and LTT should be evaluated. We would like to highlight this aspect since the existence of such a temporal association could be considered to improve the sensitivity of these *in vitro* tests thereby facilitating the detection of drug hypersensitivity.

Meanwhile, if a patient is scheduled for a skin test and BAT or LTT, it may be considered to perform the skin test first, if appropriate and not contraindicated.

## Data Availability

The original contributions presented in the study are included in the article/Supplementary Material; further inquiries can be directed to the corresponding author.
